# Supplementary material for: Changes in perceptual sampling contribute to representational drift
Source: bioRxiv. 2026 Jul 6:2026.06.24.734121. Preprint. [Version 3] doi: 10.64898/2026.06.24.734121 (PMC13370985; doi:10.64898/2026.06.24.734121)
Supplement: Supplement 1 [file NIHPP2026.06.24.734121v3-supplement-1.pdf]

bioRxiv preprint doi: https://doi.org/10.64898/2026.06.24.734121; this version posted July 6, 2026. The copyright holder for this preprint (which was not certified by peer review) is the author/funder, who has granted bioRxiv a license to display the preprint in perpetuity. It is made available under a CC-BY 4.0 International license.

changes will inevitably impact the long-term stability of neural codes.

Acknowledgements

This work was supported by the National Eye Institute under grant number **R01EY025872**.

Supporting information

Maximum mean discrepancy test details

To calculate the MMD statistic between a set of empirical and a set of resampled distance matrices, a kernel matrix K that captures the similarity between every pair of data points in these two distributions is required. Each distance matrix from the two datasets was flattened into a single vector, and the Euclidean distance between any pair of such vectors was projected into high-dimensional similarity space via a Radial Basis Function (RBF) kernel. For example, given two flattened distance matrices $\mathbf{d}_i$ and $\mathbf{d}_j$, their similarity in kernel space is calculated as:

$$K(\mathbf{d}_i, \mathbf{d}_j) = \exp\left(-\frac{\|\mathbf{d}_i - \mathbf{d}_j\|^2}{2\sigma^2}\right) \tag{7}$$

$$\sigma = \text{median}(\{\|\mathbf{d}_i - \mathbf{d}_j\| : i < j\}) \tag{8}$$

where the kernel bandwidth σ was selected to be the median Euclidean distance between all pairs of flattened distance matrices. This ensures that the scale of the kernel matrix matches that of the original data distribution, supporting optimal sensitivity when discriminating between the two samples.

After populating the kernel matrix K with all combinations of $\mathbf{d}_i$ and $\mathbf{d}_j$ from the two datasets, the unbiased MMD statistic was calculated as:

$$\widehat{MMD}^2 = \frac{1}{n(n-1)}\sum_{i\neq j}^{n} K(\mathbf{x}_i, \mathbf{x}_j) + \frac{1}{m(m-1)}\sum_{i\neq j}^{m} K(\mathbf{y}_i, \mathbf{y}_j) - \frac{2}{nm}\sum_{i=1}^{n}\sum_{j=1}^{m} K(\mathbf{x}_i, \mathbf{y}_j) \tag{9}$$

where $\{\mathbf{x}_i\}_{i=1}^{n}$ are the set of flattened distance matrices from the empirical dataset, and $\{\mathbf{y}_j\}_{j=1}^{m}$ are those from the resampled dataset (Fig S4A). To test the significance of this MMD statistic, group labels indicating whether distance matrices belonged to empirical or resampled distributions were shuffled across the combined dataset to generate 1000 permutation pairs, and the corresponding p-value was calculated based on how the original MMD statistic compares to that of these shuffled pairs containing mixed samples. The resulting p-value therefore indicates whether the distribution of empirical distance matrices is significantly distinguishable from that of the resampled.

Resampling method caveats and validation

One limitation of our primary resampling method is that we cannot preserve both the number of fixation segments and the total fixation duration to be strictly the same as the empirical data. For example, if a participant had only a single 4-second long fixation when viewing an image in one session, this fixation segment might be re-assigned to another session and concatenated with other segments to create a fixation trajectory that exceeds the 5-second trial duration. Theoretically, this doesn't affect downstream analysis, since the calculations for SGE and EMD are based on spatial

bioRxiv preprint doi: https://doi.org/10.64898/2026.06.24.734121; this version posted July 6, 2026. The copyright holder for this preprint (which was not certified by peer review) is the author/funder, who has granted bioRxiv a license to display the preprint in perpetuity. It is made available under a CC-BY 4.0 International license.

distributional properties of gaze rather than the absolute timing. However, to confirm that this caveat did not bias our statistical tests, we ran a simpler non-parametric control test by keeping the gaze content within each session intact while shuffling the session labels of each image 500 times (Fig S1A bottom). We then evaluated our empirical summary metrics against a null distribution that was derived from session-label-shuffled datasets free of fixation re-distribution and concatenations. The results remained statistically significant, confirming that the resampling artifact did not bias our primary findings (Fig S1B, S1C and Fig S2).

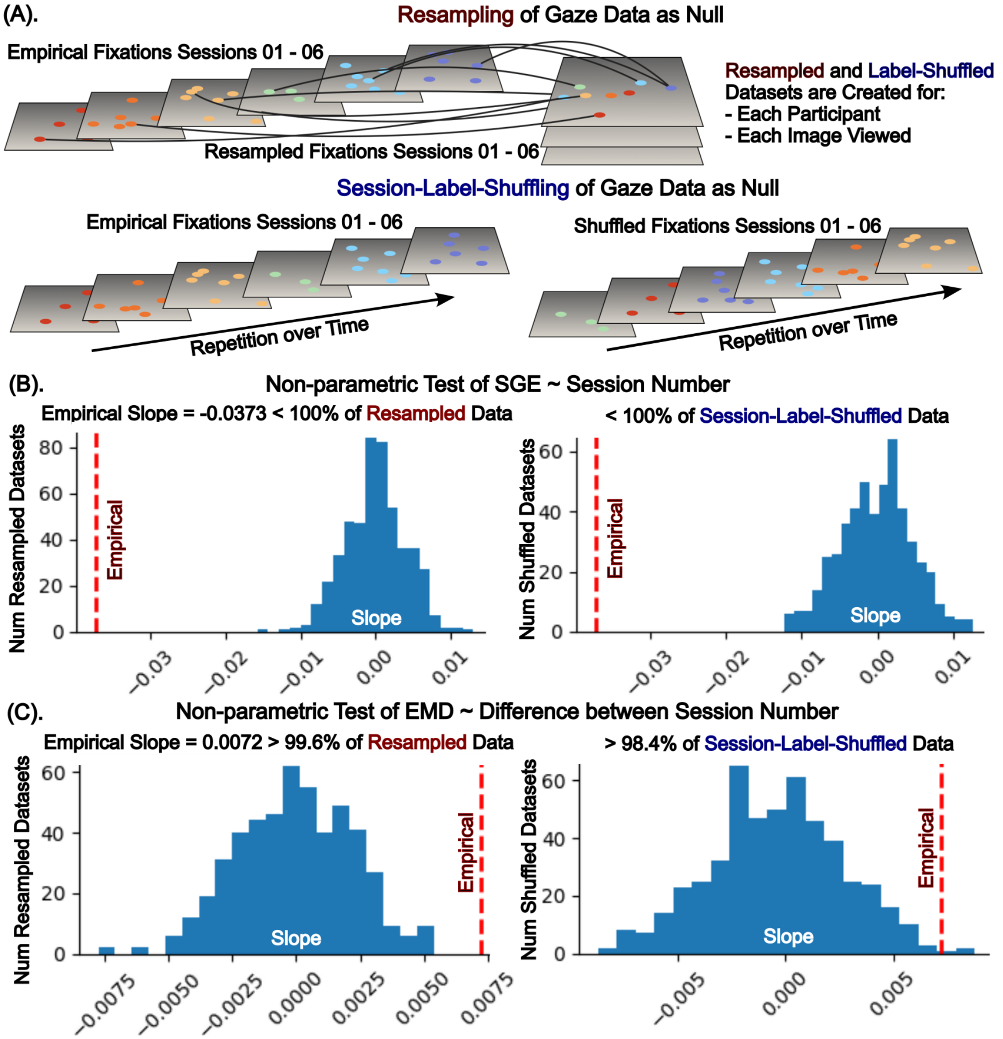

Supplementary Figure S1. Null gaze datasets do not demonstrate systematic changes in fixation profiles over time. (A): Top - chunks of fixations were randomly selected and re-distributed across different sessions to create a resampled gaze dataset. Bottom - fixation profiles were left intact while the session labels were shuffled. **(B)**: Across all resampled (left) and session-label-shuffled (right) datasets, SGE remain stable as the number of times an image was presented increased, with slopes centered around zero and consistently smaller than that of the empirical dataset. **(C)**: Across the vast majority of resampled (99.6%, left) and session-label-shuffled (98.4% right) datasets, EMD between fixation density heatmaps remain stable as the number of sessions between image presentations increased, with slopes centered around zero and smaller than that of the empirical dataset.

bioRxiv preprint doi: https://doi.org/10.64898/2026.06.24.734121; this version posted July 6, 2026. The copyright holder for this preprint (which was not certified by peer review) is the author/funder, who has granted bioRxiv a license to display the preprint in perpetuity. It is made available under a CC-BY 4.0 International license.

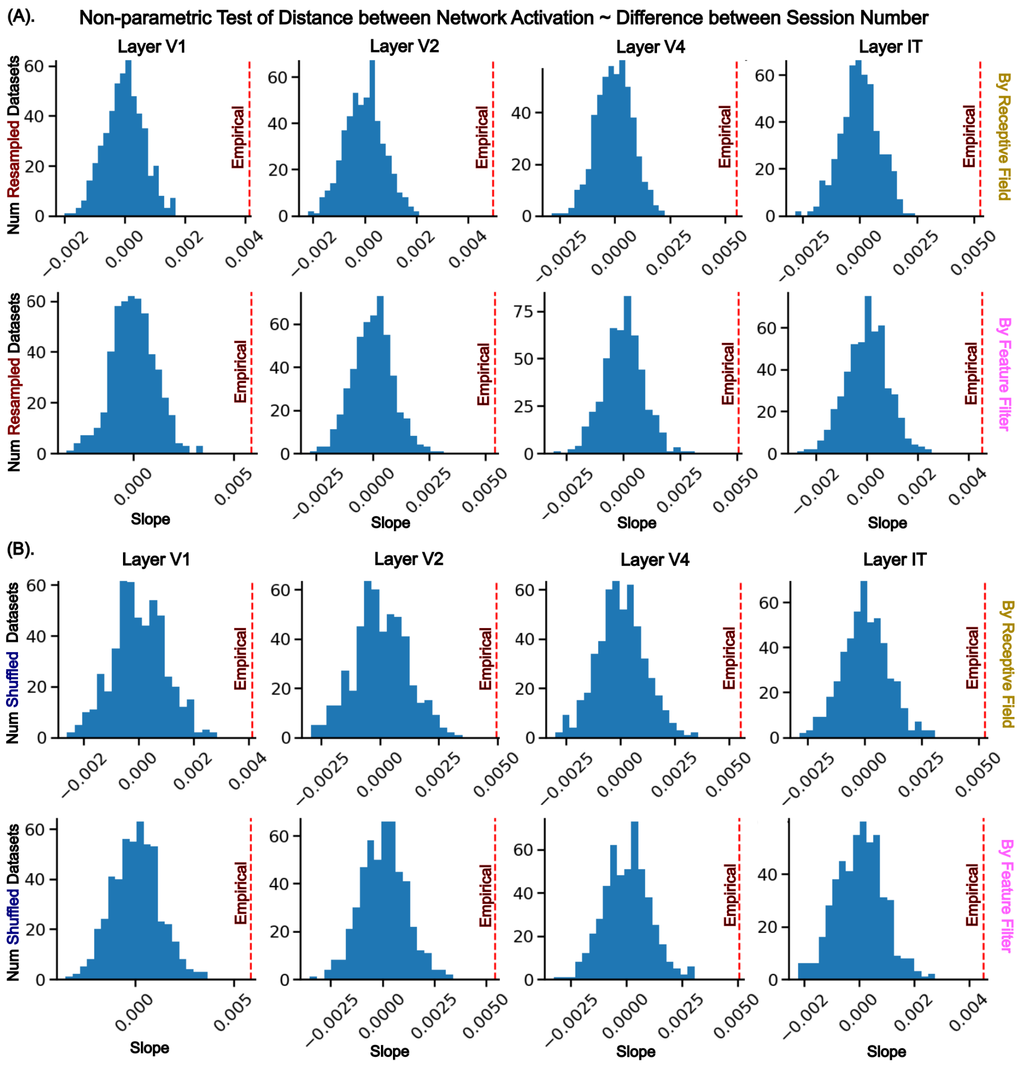

Supplementary Figure S2. Null gaze datasets do not induce representational drift-like neural patterns when passed through CORnet-S. (A): Across all resampled datasets, the dissimilarity between CORnet-S network activations remain stable as the pairs of input images were modulated with gaze data that were sampled from further separated sessions, as indicated by slopes centered around zero and consistently smaller than that of the empirical. **(B)**: The same was true for all session-label-shuffled datasets.

bioRxiv preprint doi: https://doi.org/10.64898/2026.06.24.734121; this version posted July 6, 2026. The copyright holder for this preprint (which was not certified by peer review) is the author/funder, who has granted bioRxiv a license to display the preprint in perpetuity. It is made available under a CC-BY 4.0 International license.

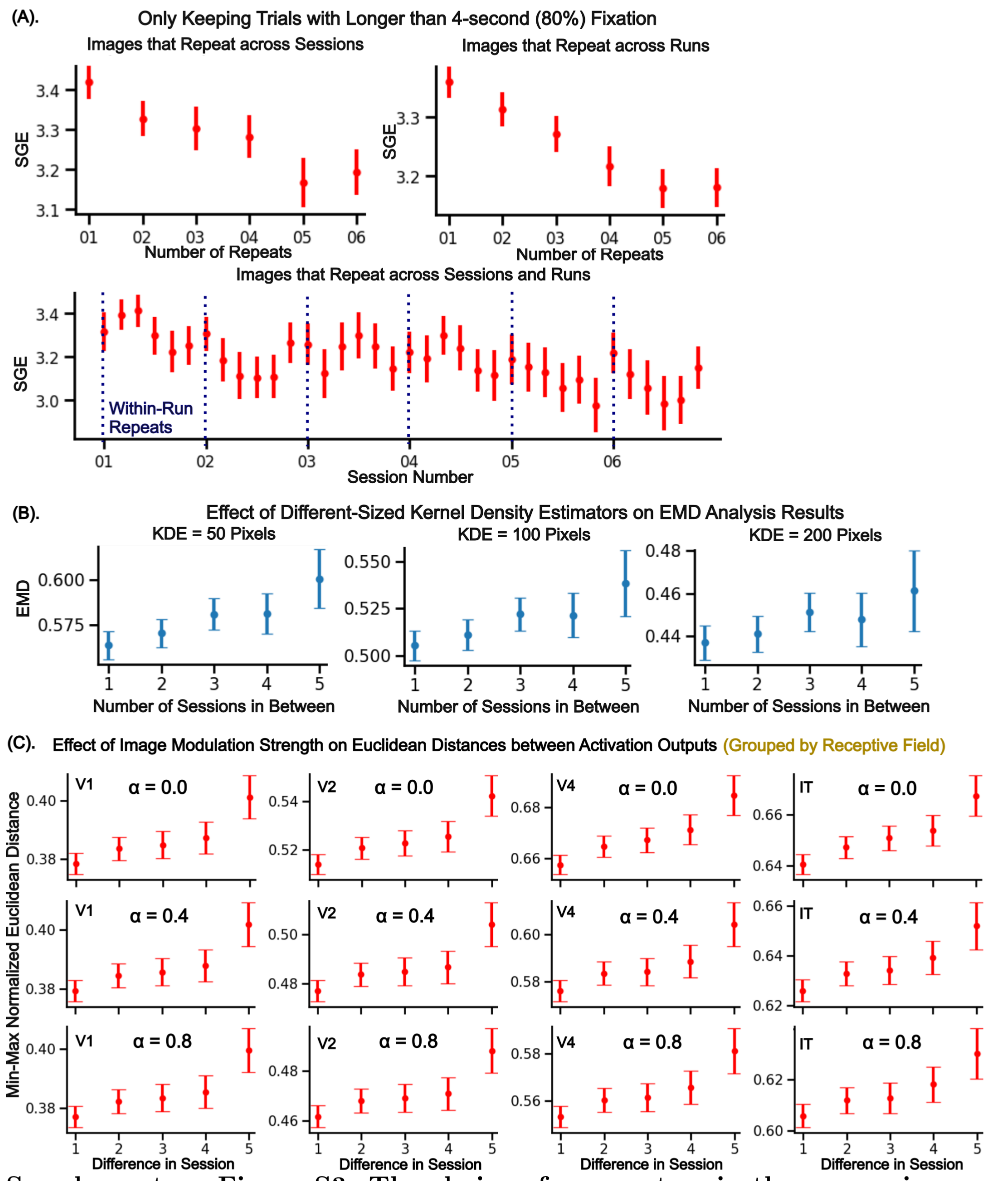

Supplementary Figure S3. The choice of parameters in the processing and analysis pipeline does not qualitatively alter the primary results. (A): SGE as a function of image repeats, excluding trials where the amount of time spent fixating was less than 80%. The markers represent mean SGE values across all 14 participants and each of the 260 images that they explored. The error bars indicate 95% confidence interval around the mean SGE. **(B)**: EMD as a function of number of sessions between image presentations, where fixation density heatmaps were created under different KDE sizes. **(C)**: Select examples of dissimilarity between CORnet-S network activations as a function of number of sessions between sampled gaze data, where the input images were modulated at different strengths according to various values of α.

bioRxiv preprint doi: https://doi.org/10.64898/2026.06.24.734121; this version posted July 6, 2026. The copyright holder for this preprint (which was not certified by peer review) is the author/funder, who has granted bioRxiv a license to display the preprint in perpetuity. It is made available under a CC-BY 4.0 International license.

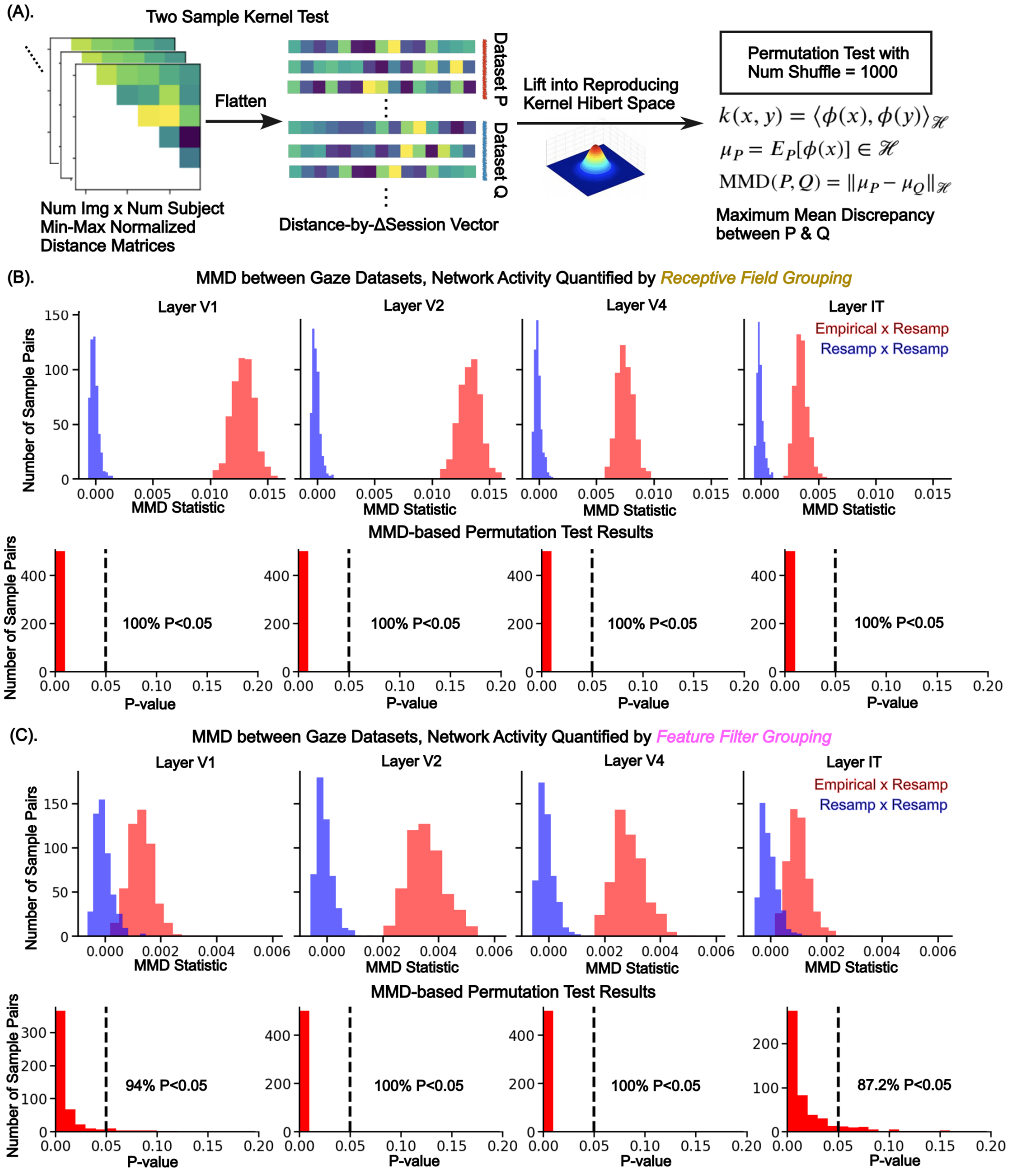

Supplementary Figure S4. MMD-based two-sample permutation test. (A): Schematic of the testing procedure. **(B)**: For network activation distance matrices quantified via grouping units by their receptive fields, the MMD between those derived from the empirical and the resampled null datasets (top red), as well as the permutation test results with 1000-fold shuffling (bottom). **(C)**: Same as **B** but for network activation distance matrices quantified via grouping units by their feature selectivity profiles.
